# A toolkit to rapidly modify root systems through single plant selection

**DOI:** 10.1186/s13007-021-00834-2

**Published:** 2022-01-10

**Authors:** Charlotte Rambla, Sarah Van Der Meer, Kai P. Voss-Fels, Manar Makhoul, Christian Obermeier, Rod Snowdon, Eric S. Ober, Michelle Watt, Samir Alahmad, Lee T. Hickey

**Affiliations:** 1 Queensland Alliance for Agriculture and Food Innovation, The University of Queensland, St Lucia, QLD 4072 Australia; 2 Department of Plant Breeding, IFZ Research Centre for Biosystems, Land Use and Nutrition, Justus Liebig University, Heinrich-Buff-Ring 26-32, 35392 Giessen, Germany; 3 National Institute of Agricultural Botany (NIAB), 93 Lawrence Weaver Road, Cambridge, CB3 0LE UK; 4 School of BioSciences, Faculty of Science, University of Melbourne, Parkville, VIC 3010 Australia

**Keywords:** Root traits, Seminal root angle, Root biomass, Wheat breeding, Root system, Segregating populations, Speed breeding, Single plant selection

## Abstract

**Background:**

The incorporation of root traits into elite germplasm is typically a slow process. Thus, innovative approaches are required to accelerate research and pre-breeding programs targeting root traits to improve yield stability in different environments and soil types. Marker-assisted selection (MAS) can help to speed up the process by selecting key genes or quantitative trait loci (QTL) associated with root traits. However, this approach is limited due to the complex genetic control of root traits and the limited number of well-characterised large effect QTL. Coupling MAS with phenotyping could increase the reliability of selection. Here we present a useful framework to rapidly modify root traits in elite germplasm. In this wheat exemplar, a single plant selection (SPS) approach combined three main elements: phenotypic selection (in this case for seminal root angle); MAS using KASP markers (targeting a root biomass QTL); and speed breeding to accelerate each cycle.

**Results:**

To develop a SPS approach that integrates non-destructive screening for seminal root angle and root biomass, two initial experiments were conducted. Firstly, we demonstrated that transplanting wheat seedlings from clear pots (for seminal root angle assessment) into sand pots (for root biomass assessment) did not impact the ability to differentiate genotypes with high and low root biomass. Secondly, we demonstrated that visual scores for root biomass were correlated with root dry weight (r = 0.72), indicating that single plants could be evaluated for root biomass in a non-destructive manner. To highlight the potential of the approach, we applied SPS in a backcrossing program which integrated MAS and speed breeding for the purpose of rapidly modifying the root system of elite bread wheat line Borlaug100. Bi-directional selection for root angle in segregating generations successfully shifted the mean root angle by 30° in the subsequent generation (*P* ≤ *0.05*). Within 18 months, BC_2_F_4_:F_5_ introgression lines were developed that displayed a full range of root configurations, while retaining similar above-ground traits to the recurrent parent. Notably, the seminal root angle displayed by introgression lines varied more than 30° compared to the recurrent parent, resulting in lines with both narrow and wide root angles, and high and low root biomass phenotypes.

**Conclusion:**

The SPS approach enables researchers and plant breeders to rapidly manipulate root traits of future crop varieties, which could help improve productivity in the face of increasing environmental fluctuations. The newly developed elite wheat lines with modified root traits provide valuable materials to study the value of different root systems to support yield in different environments and soil types.

**Supplementary Information:**

The online version contains supplementary material available at 10.1186/s13007-021-00834-2.

## Background

The root system of a wheat (*Triticum aestivum L.*) crop plays an essential role in anchorage and uptake of water and nutrients required for photosynthesis and growth. Despite the importance of below-ground traits, wheat breeding programs over the past 100 years have largely focussed on direct selection and improvement of above-ground traits. Wheat root system architecture is typically governed by many genes with small effect, often with a degree of epistasis or complex interactions that may change according to environmental conditions [1, 2]. This complexity restrains our understanding of the genetic controls and the value of specific root traits in different environments [3, 4]. Furthermore, while methods have been developed to evaluate root system architecture in the field [5–8], screening large populations is resource-intensive and challenging due to the heterogeneous nature of soil.

Controlled environment techniques have been developed to enable the identification of more heritable root traits and reproducible phenotyping results compared to field conditions [9]. A number of seminal root phenotyping systems in controlled conditions have been developed, including the ‘clear pot’ method [10], which was firstly used for direct selection of seminal root angle in segregating wheat populations [11] and has since been successfully applied to durum wheat [12] and barley [13]. Seminal root angle is a simple root trait to phenotype at the seedling stage [1, 14] and in some studies has been associated with the three-dimensional growth and functioning of the root system later in the season [15, 16]. However, in a comprehensive study by Rich et al. [17], seedling root traits assayed using several phenotyping techniques under controlled conditions were compared to root traits measured in the field, and inconsistent correlations were found. Results varied between seedling and mature root traits across trials and seasons, showing context dependency and plasticity of root trait phenotypes. Watt et al. [18] reported a significant correlation between primary root traits using rolled paper tubes and root traits at two and five leaf stages in the field (r^2^ = 0.63 and 0.79, respectively). However, a correlation with mature roots at anthesis was not observed. Nevertheless, seminal root phenotyping approaches have been widely used in controlled conditions allowing morphological and physiological traits to be measured out of season. These screening techniques can be applied to develop populations enriched with desired root traits for subsequent field evaluation. By applying cost-effective screening and selection in early generations more labour intensive and expensive field testing can be performed in later generations using a smaller set of elite materials [19].

While functional phenomics pipelines in control environments can help identify and prioritise the study of key root traits [20], incorporating the traits into elite germplasm is a slow process. For instance, 5–10 years of pre-breeding was needed to incorporate root traits for a range of soil constraints into advanced germplasm [11]. MAS can reduce the reliance on phenotyping and aid the selection of key genes or QTLs associated with a range of root traits. In rice, *DEEPER ROOTING 1* (*DRO1*) was successfully backcrossed into an elite shallow rooting cultivar using a linked molecular marker [21]. A key limitation for applying this approach in wheat and many other crop species is a lack of markers associated with QTL that have a substantial effect on root system architecture [16]. Furthermore, most QTL mapping studies in wheat have relied on root phenotyping under controlled conditions, thus knowledge of how these effects translate to field environments is limited.

There is a need to develop new approaches to speed up research and pre-breeding programs targeting root traits that could improve yield in different environments [11, 22]. Spring wheat speed breeding platforms that facilitate rapid generation advance are helping to fast-track pre-breeding efforts and enable the introgression of new traits into elite materials within 1–2 years [23]. Methods that enable trait screening and selection during the speed breeding process have been developed for several disease resistance traits [24, 25]. We envisage an opportunity to exploit the speed breeding system to accelerate root trait introgression. To enable this, methods are needed to screen large segregating populations for root morphological traits, where individual plants displaying desirable phenotypes can be selected and promoted for generation advance or backcrossed. Richards and Passioura [26] employed a similar approach, backcrossing two Australian commercial wheat varieties with a landrace chosen as a donor of the narrow xylem vessel trait. The BC_1_F_2_ populations resulted in reduced xylem vessel diameter from 65 to less than 55 µm. Selection for narrow xylem vessels increased yield between 3 and 11% suggesting that introgressing beneficial root traits may have a large impact on yield improvement.

Here we report a rapid non-destructive method to enable SPS for root traits in wheat, specifically seminal root angle and root biomass. The approach combines both phenotypic selection and MAS, along with speed breeding to rapidly introgress the root traits into elite germplasm. This provides a useful framework for pre-breeding and research programs seeking to rapidly modify root systems and study the value of specific root traits in elite germplasm.

## Methods

### Plant materials

A panel of spring wheat lines was evaluated to determine the feasibility of screening single plants for both seminal root angle and root biomass. The panel included two International Maize and Wheat Improvement Center (CIMMYT) varieties (Kingbird and Borlaug100), two Australian commercial varieties (Suntop and Mace) and 13 accessions from a diversity panel studied by Voss-Fels et al. [27]. Borlaug100 was selected as the recipient background to introgress key root traits. It is a high-yielding wheat which was developed at CIMMYT and first imported into Australia in 2015 via the CIMMYT-Australia-ICARDA Germplasm Evaluation (CAIGE) project. The six accessions from the diversity panel have known root biomass phenotypes and haplotypes for the root biomass QTL on chromosome 5B [28]. Three of the six accessions were selected as donor parents for root trait introgression: SW107 and SW388 for high root biomass (both positive for the 5B QTL), and SW309 for low root biomass (negative for the 5B QTL).

### Testing the ability to integrate seminal root angle and root biomass screening protocols

Two experiments were conducted to determine the feasibility of screening individual wheat plants for both seminal root angle and root biomass. The goal was to develop a non-destructive method suitable for SPS, which integrated two established protocols: (1) the ‘clear-pot’ method [10], which enables phenotyping for seminal root angle through image analysis, and (2) a hydroponic sand-based system [27], which allows efficient root washing to phenotype root dry biomass. However, the root dry biomass phenotyping method typically involves root and shoot dissection and drying, resulting in plant destruction. To integrate these methods, firstly, seminal root angle screening using the clear pot method was performed and selected plants were transplanted into sand-filled pots to grow-on for subsequent root biomass assessment. Next, visual assessment of biomass was recorded for washed intact root systems, and selected plants were transplanted and grown-on to enable generation advance or crossing using selected individual plants directly. Prior to applying this method to segregating wheat populations, it was established through experiments that root biomass phenotypes were not compromised during the transplanting process, and root biomass screening could be faithfully conducted visually rather than destructively.

#### Comparison of root biomass phenotypes: direct sowing versus transplants

Two genotypes were selected to test whether wheat seedlings could be transplanted from clear pots into sand pots for root biomass assessment. SW300 was included as the low root biomass standard (lacks the 5B QTL for high biomass), whereas SW411 was included as the high root biomass standard (carries the 5B QTL for high root biomass). Seeds were initially sown into clear pots for seminal root angle assessment, as described by Richard et al. [10]. A total of 24 seeds per line were sown across two 4 L clear pots (ANOVApot^®^, 200 mm top diameter, 190 mm height, http://www.anovapot.com/php/anovapot.php) adopting a randomized complete block design (RCBD). Clear pots were filled with a pine bark potting media (70% composted pine bark 0–5 mm, 30% coconut peat, pH 6.35, EC = 650 ppm, nitrate = 0, ammonium < 6 ppm and phosphorus = 50 ppm), and seeds were sown using tweezers by carefully placing the seed vertically, at a depth of 2 cm and every 2.5 cm with embryo downwards and facing the pot wall to facilitate root growth along the transparent wall. After sowing, the clear pots were placed inside 4 L black pots (ANOVApot^®^, 200 mm diameter, 190 mm height) to block light from reaching the developing roots. Pots were fully watered before sowing and were not watered until after imaging. Plants were grown in the glasshouse at a constant temperature (17 ± 2 °C) over 24 h with diurnal (12 h) natural light. Imaging of seminal root angle was performed at five days after sowing, using a digital camera (Canon PowerShot SX600 HS 16MP Ultra-Zoom) and seminal root angle for each plant, the angle (α) between the first pair of seminal roots, was measured at approximately 3 cm distance from the seed using software (ImageJ) (http://imagej.nih.gov/ij/).

After the imaging step, seedlings were transplanted into pots filled with sand for subsequent root biomass phenotyping, as described by Voss-Fels et al. [27]. Each 1.4 L ANOVA pot (ANOVApot^®^, 137 mm diameter, 140 mm height) was filled with approximately 1.7 kg of coarse washed sand (particle size ranging 0.075–4.75 mm) to facilitate root washing. With gentle water flow, roots could be easily and cleanly separated from the sand, which minimised damage to roots. The experimental design consisted of 16 pots with two plants of the same genotype in each pot, placed inside a clear plastic storage container (65 cm length and 35 cm width with a capacity of 36 L), allowing eight replications per genotype. Containers were fitted with capillary mats to ensure water and nutrient uptake and hand-watered daily using a commercial hydroponic solution with complete nutrients (Cultiplex Extra-Nutrex Grown). The hydroponic solution was diluted to adjust the nutrient concentrations so that the growth requirements of the plants were met as they developed (days 1–10: 1.50 mL/L, days 11–17: 2 mL/L, days 18–21: 2.50 mL/L).

At the time of transplanting, a second root biomass experiment was initiated, where seeds were sown directly into sand pots. The transplant and direct-seeded experiments were performed simultaneously, adopting a similar design and layout. The plants in both assays were grown in the same temperature-controlled glasshouse set to 22/17 °C (day/night) under natural (12 h) photoperiod. The experiments were designed to assess the impact of transplanting root biomass and on the ability to accurately differentiate high and low root biomass phenotypes. At 21 days after transplanting and direct sowing, plants were extracted with minimum root disruption by placing the pot in a bucket of water and washing off the sand with clean water. Roots and shoots were separated using scissors, roots were placed in a dehydrator at 65 °C for 72 h, then weighed. A Tukey’s test was performed to determine differences in root dry biomass within and between the experiments using the corrected multiple comparison method with a confidence interval of 95% and an error rate of 5%, using the R package ‘*agricolae*’ (software Version 4.0.2, R Core team 2020).

#### Evaluating the ability to perform non-destructive visual assessment of root biomass

The protocol for phenotyping root biomass reported by Voss-Fels et al. [27] is destructive, as roots must be dissected from the shoot before weighing, making it impossible to use plants with desirable phenotypes for further crossing. Therefore, non-destructive visual scoring of the size of the root system was assessed, which could serve as a surrogate for root biomass. Seeds of 17 genotypes (Additional file 1: Table S1 summarizes relevant traits of all the lines screened) were sown directly into sand-filled pots (as described above), using six replicate pots per genotype, and four plants per pot (total of 404 plants). A total of 102 pots were arranged according to a RCBD design across six containers (i.e., 17 pots/container). Plants were watered daily using the hydroponic solution as described above.

**Fig. 1:**
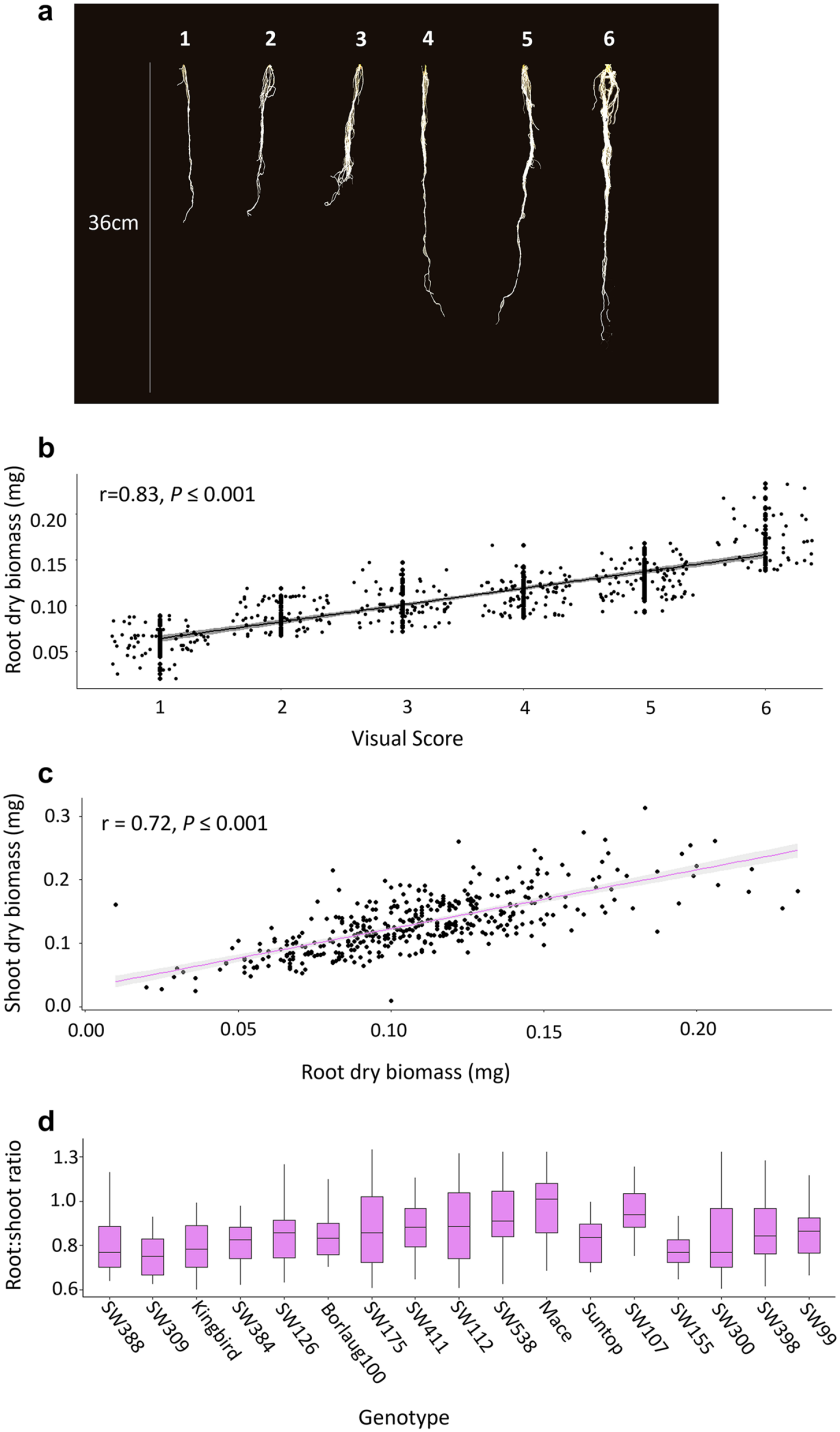
Summary of root and shoot biomass data for the panel of 17 lines used for visual scoring to estimate root biomass non-destructively. This panel includes Borlaug100 and the three donors (SW107, SW309 and SW388) used to develop introgression lines with different root configurations, where **a** displays the 1–6 scale used for ‘visual assessment method’ of root biomass. The 1–6 scale: 1 = very fine root system with short root length and very few surface roots, 2 = fine root system, short root length and few surface roots, 3 = fine root system, short root length and some surface roots, 4 = intermediate root system, long root length and intermediate surface roots, 5 = strong root system, long root length and strong surface roots, and 6 = strong root system, long root length and strong surface roots with nodal roots clearly visible. **b** Shows the relationship between visual scores and root dry biomass, **c** Shows the relationship between root dry biomass and shoot dry biomass, and **d** presents boxplots for root:shoot ratio

Roots were washed 21 days after sowing and were arranged on a clear flat surface to facilitate visual scoring. The size of the root system for each plant was selected with a ‘Visual assessment method’ that consisted in visually assessing the roots using a scale of 1–6, where 1 = very fine root system with short root length and very few surface roots, 2 = fine root system, short root length and few surface roots, 3 = fine root system, short root length and some surface roots, 4 = intermediate root system, long root length and intermediate surface roots, 5 = strong root system, long root length and strong surface roots, and 6 = strong root system, long root length and strong surface roots with nodal roots clearly visible (Fig. 1a). To minimise error and variability, visual scoring was performed by the same person. Prior to scoring, an assessment for a full range of phenotypes was performed and used as an ‘eye-adjustment’. After root scoring, roots were separated from the stem tissue above the crown at 26 days after sowing and both sections were placed in a forced-air dehydrator at 65 °C for 72 h.

Dry weight of root and shoot biomass was recorded using a scale (AND, HR-200 scales) with 0.0001 g accuracy. The reliability of visual scoring for root biomass was examined through a Pearson’s correlation coefficient with the actual root dry biomass (Fig. 1b).

The relationship between root dry biomass and shoot dry biomass was also explored to determine if selection targeting root biomass would result in indirect selection for shoot biomass (Fig. 1c). Furthermore, to investigate the potential genetic variation in root-shoot biomass configurations, root:shoot ratio (R:S) was calculated for each of the 17 genotypes. Following ANOVA, a Fisher’s least significant difference (LSD) test was conducted to compare the means to detect differences between genotypes with a 95% family-wise confidence interval with the function *LSD.test* using agricolae in R (software Version 4.0.2, R Core team 2020).

### Overview of the single plant selection (SPS) approach for root trait introgression

**Fig. 2:**
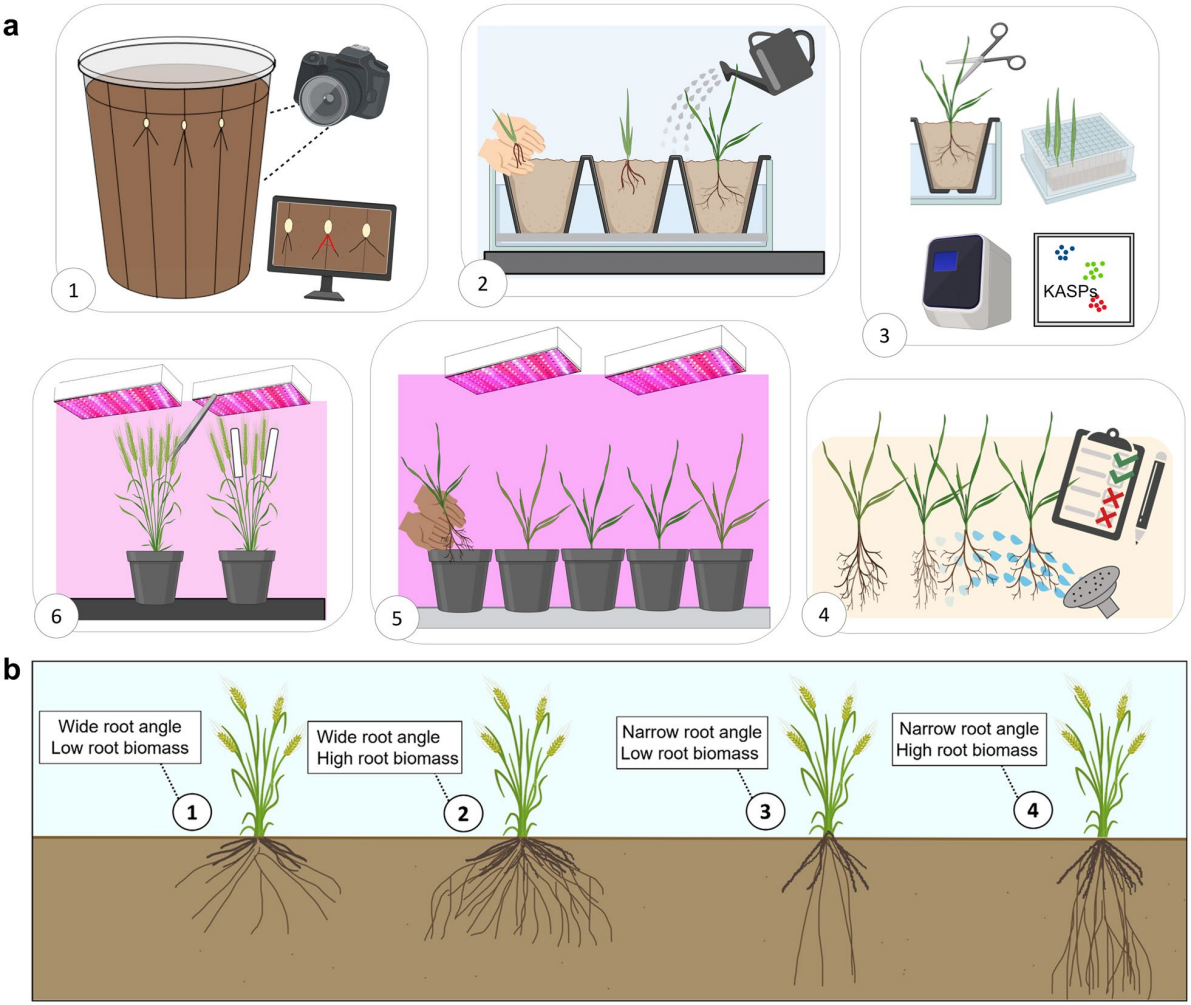
**a** An overview of the non-destructive single plant selection (SPS) approach to create elite varieties with ‘designer’ roots, which includes integration of marker-assisted selection (KASP) for the root biomass QTL on chromosome 5B and backcrossing under speed breeding. SPS involves six key steps: **1** seminal root angle screening and selection of single plants using the clear pot method [10], **2** a semi-hydroponic assay where the plants are transplanted into sand as detailed in [44], **3** plants are genotyped using KASP markers associated with major QTL, **4** roots are washed and scored using the 1–6 scale, and plants with high and low root biomass are selected using a combination of visual scores and KASP marker data, **5** Selected plants are then transplanted into potting mix and grown under speed breeding conditions, and **6** plants are backcrossed or selfed for line development. **b** The four root system configurations which could be assembled by targeting seminal root angle and root biomass traits: **1** wide angle-high root biomass, **2** wide angle-low root biomass, **3** narrow angle-high root biomass, and **4** narrow angle-low root biomass

A visual summary of the six key steps involved in the selection pipeline is provided in Fig. 2a. This process integrates non-destructive phenotypic screening for seminal root angle and root biomass, MAS for a major root biomass QTL, and backcrossing under speed breeding to accelerate plant development. This approach was used to rapidly generate elite introgression lines using Borlaug100 as the recipient background. Selection aimed to create introgression lines with four different root trait configurations (Additional file 1: Fig. S1; Fig. 2b): wide angle-high root biomass, wide angle-low root biomass, narrow angle-high root biomass and narrow angle-low root biomass. A summary of each step is provided below, and a detailed list of materials used for SPS with corresponding descriptions are provided in Additional file 2.

#### Step 1—seminal root angle screening

The SPS approach started with assessment of a large segregating population (BC_1_F_2_; total 968 plants; Additional file 1: Fig. S2) for seminal root angle using the clear pot method, as per Richard et al. [10]. Five days after sowing the seminal roots were imaged (Canon PowerShot SX600 HS 16MP Ultra-Zoom) and seminal root angle measured using ImageJ software (http://imagej.nih.gov/ij/). Individual plants representing the population tails or extreme root angle phenotypes were selected (160 plants), including both narrow and wide root angles. Pots were fully watered before sowing and were not watered until after imaging. Plants were grown in the glasshouse at a constant temperature (17 ± 2 °C) over 24 h with diurnal (12 h) natural light.

#### Step 2—transplanting into sand (semi-hydroponic sand-based system)

The selected plants were carefully extracted from clear pots and transplanted into sand-filled pots (two plants per pot) for root biomass assessment. Pots were placed into containers placed on capillary mats; 15 pots were placed in each container in an RCBD design. Plants were grown in the glasshouse at a constant temperature (17 ± 2 °C) over 24 h with diurnal (12 h) natural light. Plants were watered daily using hydroponic solution (1.50 mL of Cultiplex Extra-Nutrex Grown per litre of water); concentrations were slowly increased according to plant growth: days 1–10: 1.50 mL/L, days 11–17: 2 mL/L, days 18–21: 2.50 mL/L.

#### Step 3—KASP marker screening

Leaf tissues were sampled from wheat plants at the seedling stage to ensure quality DNA was extracted. Four pieces of 3 cm long leaf tissue were placed in 1.2 mL cluster tube (96-tube racks) and freeze-dried for 48 h prior to dispatchment to collaborators at the Department of Plant Breeding, Justus Liebig University, Giessen, Germany. Samples were then genotyped using the high-quality extracted DNA and genotypic data were obtained to assist in selecting individuals for crossing. Selection for root biomass was based on KASP markers developed for the major QTL reported on chromosome 5B [27]. Three robust KASP assays (*HapB3-2, HapB6-1* and *HapA2-2*) for the 5B locus were developed by Makhoul et al. [28] to distinguish the haplotype combination associated with high root biomass from other haplotype combinations associated with low root biomass [27]. The high biomass trait is associated with ‘T’ allele for marker *BS00029852_51* and ‘T’ allele for marker *Tdurum_contig48959_1172* in haploblock b and with ‘T’ allele for marker *Excalibur_c25522_755* in haploblock a.

#### Step 4—root biomass scoring and selection

Twenty-one days after transplanting, the plants were extracted with minimum disruption to the roots and the sand was washed off by placing the pot in a bucket of clean water. Following root washing, all the plants within the same category of root angle were lined up based on the numbering of the pots on a clear surface. Root biomass for each plant was scored using the ‘Visual assessment method’. A number of 32 plants representing the population tails or extreme root biomass phenotypes were selected, including both high and low root biomass. Prior to scoring, an assessment for a full range of phenotypes was performed and used as an ‘eye-adjustment’ where recurrent parent and donor lines were scored first, followed by the progenies. Further selection within the tails was applied based on the KASP marker results. This ensured that individuals selected for high root biomass displayed both superior phenotypes and carried the desirable allele for the 5B QTL region. To capture other loci that could be important for trait expression, individuals which lacked the QTL but displayed high root biomass were also retained. Hence, to increase the confidence and accuracy of selection, a combination of both MAS and phenotypic selection was employed.

#### Step 5 and step 6—growing-on selected plants for backcrossing or line development

Finally, the selected plants (32 plants) carrying the desired combinations (Fig. 2b: wide-high root biomass, wide-low root biomass, narrow-high root biomass and narrow-low root biomass) of root traits were transplanted into potting mix and grown-on under speed breeding conditions [29] to accelerate plant development and enable rapid backcrossing or selfing for line development.

### Development of introgression lines with different root configurations

The three donor lines (SW107, SW388 and SW309) for high and low root biomass were crossed to Borlaug100 to create F_1_ seeds. An overview of the crossing scheme is provided in Additional file 1: Fig. S2. The F_1_ plants were backcrossed to the recurrent parent, and the BC_1_F_1_ plants were self-pollinated to produce large segregating (BC_1_F_2_) populations. To accelerate population development, all generations were grown under speed breeding conditions at UQ glasshouse facilities [29]. The SPS approach outlined in Fig. 2 was applied to the BC_1_F_2_ populations to select individual plants so that all four root system configurations were represented. The phenotypic screening process was repeated for every consecutive plant generation to increase the homozygosity for root traits in the selected lines. A Tukey’s test was performed to determine differences between the mean of each density distribution of the population (narrow and wide tails) and the recurrent parent.

### Investigating the effectiveness of single plant selection in segregating populations

The progenies from BC_2_F_2_ plants were selected for ‘narrow’ and ‘wide’ seminal root angles by growing the BC_2_F_2_:F_3_ populations and screening for seminal root angle. The population distributions for both ‘wide’ and ‘narrow’ groups were compared with the recurrent parent.

A Pearson’s correlation coefficient was calculated for the BC_2_F_2_:F_3_ populations for seminal root angle and root biomass derived by visual score, to further explore the relationship between the two root traits. A total of 120 BC_2_F_3_:F_4_ lines, along with the recurrent parent and donor lines, were evaluated for above-ground traits under field conditions in 2020 at The University of Queensland Gatton Research Farm, Gatton, Queensland, Australia (27°33′4ʺ S, 152°16′32ʺ E). The lines were sown in single 6 m long rows and key agronomic traits were recorded. Based on plant height and flowering time data collected, a total of 20 introgression lines were selected for yield evaluations in 2021 (Additional file 1: Table S3). This strong selection for flowering time and plant height, ensured that the introgression lines displayed a high degree of similarity to their recurrent parent for these above-ground traits. The 20 selected introgression lines were re-genotyped with KASP markers to confirm the QTL status and the lines were also phenotyped for root traits (seminal root angle and root biomass) under controlled conditions using the SPS method elucidated above. Root phenotypes displayed by introgression lines were compared to the recurrent parent Borlaug100 using a Fisher-LSD test to determine significant differences.

## Results

### Seminal root angle and root biomass screens were combined using a non-destructive approach

**Fig. 3:**
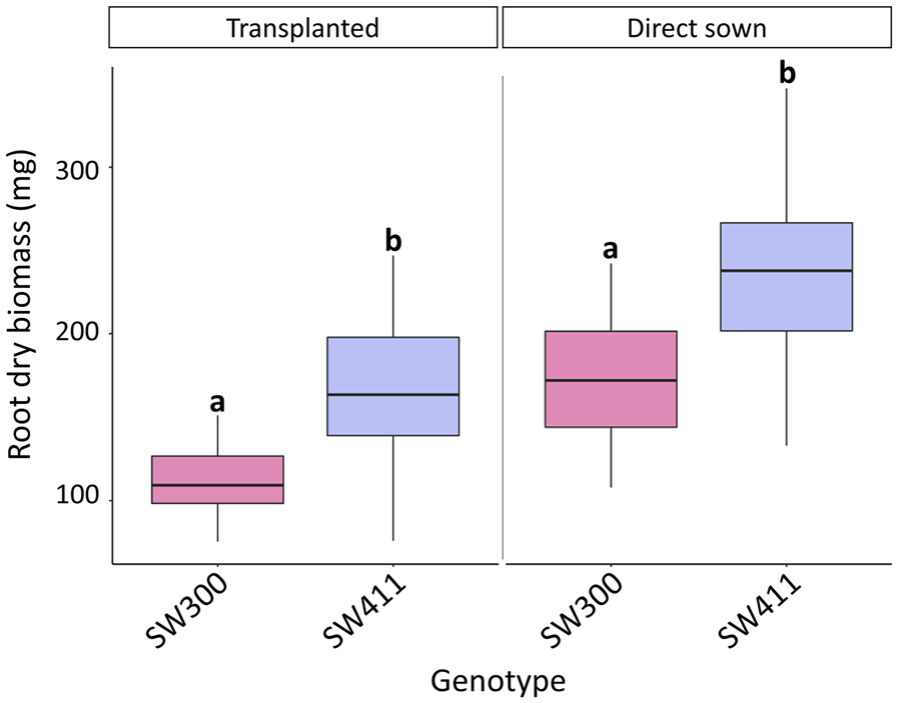
Boxplot displaying root dry biomass for transplanted and directly sown plants obtained for high (SW411) and low (SW300) standards. Means for lines labelled with the same letter are not significantly different according to Tukey’s multiple comparisons of means test (*P* ≥ 0.05) within treatments

To perform non-destructive SPS for both root traits, it was crucial to confirm that genotypes with high and low root biomass could be differentiated when seedlings were transplanted from clear pots into sand pots. On average, root dry biomass measurements were higher for plants that were directly sown into sand. For example, root dry biomass for transplanted SW411 was 164.9 mg versus 237.6 mg for directly sown seed; and dry root biomass for transplanted SW300 was 111.4 mg in comparison to the same genotype directly sown seeds 172.9 mg. Importantly, transplanting did not affect the ability to differentiate high and low root biomass genotypes, as the low root biomass standard (SW300) displayed significantly lower root biomass in comparison to the high root biomass standard (SW411) in both treatments (*P* ≤ 0.001; Fig. 3).

There was a very strong correlation between root dry biomass and visual scores (r = 0.83; *P* ≤ 0.001; Fig. 1b; Additional file 1: Fig. S3a). Notably, a strong correlation was observed between root and shoot dry biomass (r = 0.72; *P* ≤ 0.001; Fig. 1c; Additional file 1: Fig. S3b).

### Key parental lines displayed genetic variation for root:shoot ratio

There was significant variation for R:S in the panel, including some of the lines used for developing introgression lines (Borlaug100, SW107 and SW309; Fig. 1d; Additional file 1: Table S1). For instance, the two donor lines SW309 (0.76) and SW107 (0.97) displayed different R:S ratios (Fig. 1d). Among the 17 genotypes measured, the lowest R:S ratio was Suntop (0.75), and the highest was Mace (0.99), demonstrating a high degree of variation in total carbon allocation to roots.

### Single plant selection for seminal root angle shifted population distribution

**Fig. 4:**
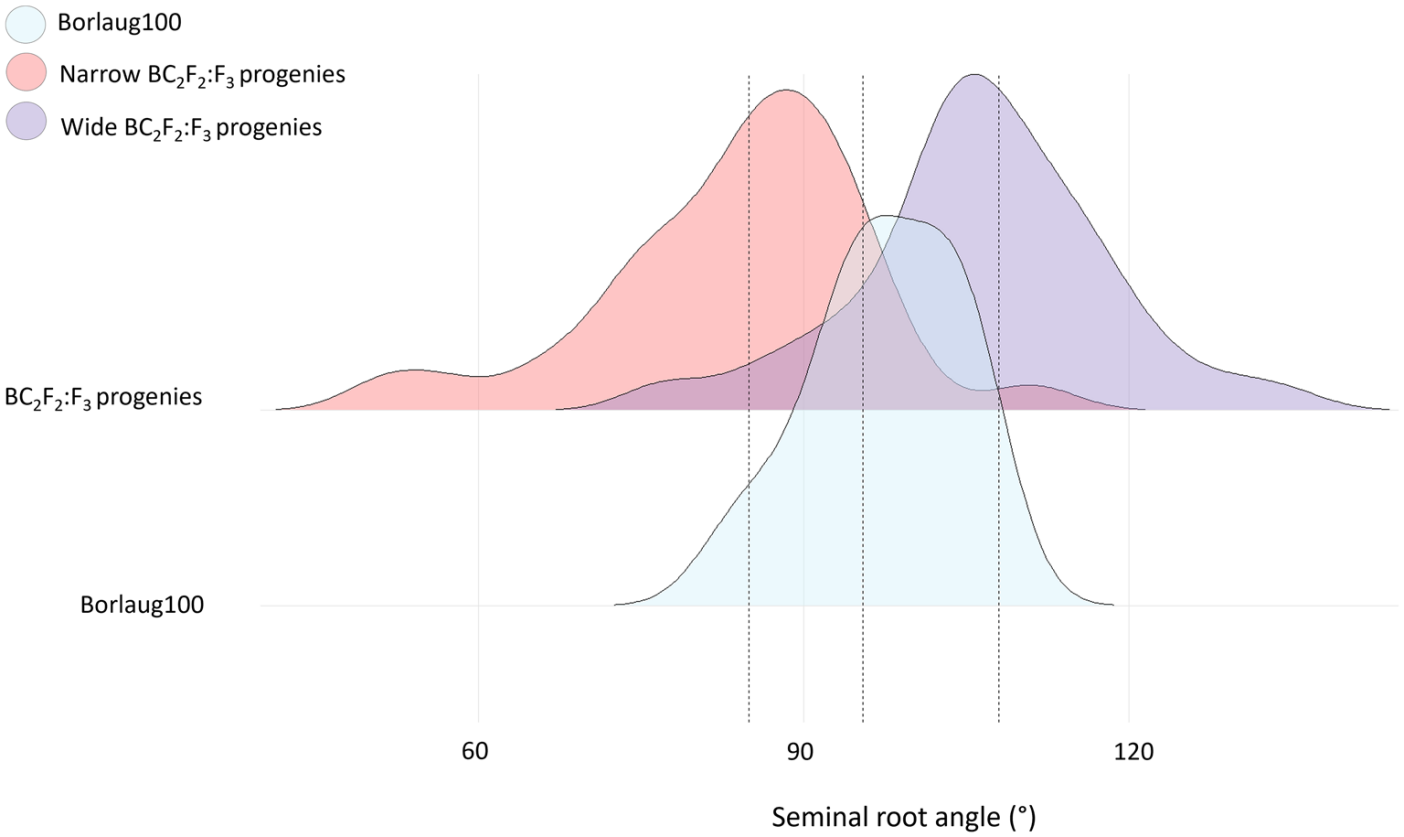
Density distribution for root angle displayed by BC_2_F_2_:F_3_ populations selected for ‘narrow’ (64 plants) and ‘wide’ (64 plants) phenotypes in the previous generation (BC_2_F_2_). BC_2_F_2_:F_3_ derived from crossing Borlaug100 and three donors (SW107, SW388 and SW309). Distribution of recurrent parent Borlaug100 (16 plants) is also displayed. The dotted vertical lines indicate the mean of the narrow tail (73.3°), wide tail (104.7°) and the recurrent parent Borlaug100 (85.8°). Significant differences were revealed between the means of Borlaug100 and the narrow tail (P ≤ 0.001), Borlaug100 and the wide tail (P ≤ 0.001), narrow and wide tails (P ≤ 0.001)

The response to selection applied to seminal root angle in the BC_2_F_2_ generation was observed the following generation (i.e., BC_2_F_2:_F_3_)_._ Evaluation of the BC_2_F_2:_F_3_ progeny representing the narrow and wide tails in comparison to the recurrent parent (Borlaug100) revealed significant differences (Borlaug100—Narrow tail *P* ≤ 0.001, Borlaug100—Wide tail *P* ≤ 0.001, Narrow-Wide *P* ≤ 0.001) in seminal root angle phenotypes (Fig. 4). On average, the narrow tail obtained a mean of 73.3°, the wide tail obtained a mean of 104.7°, and the recurrent parent Borlaug100 obtained a mean of 85.8°.

### Correlations between root traits were specific to trait configurations

The correlation between seminal root angle and visual root biomass scores for the entire BC_2_F_2:_F_3_ population, including all root trait configurations (i.e., narrow seminal root angle-high root biomass; narrow seminal root angle low root biomass; wide seminal root angle-high root biomass and wide seminal root angle-low root biomass) was r = 0.17 (*P* = 0.07). However, within the different configurations, the strength of correlations between root angle and biomass varied. For example, for the single configuration ‘narrow’ and ‘low root biomass’ the correlation was significant (r = 0.42, *P* = 0.02). On the other hand, for the category ‘wide’ and ‘high root biomass’ the correlation was not significant (r = 0.24; *P* = 0.19). Broad and narrow-sense heritability were also calculated for both traits, where seminal root angle showed higher heritability (H^2^ = 0.445 and h^2^ = 0.374) over visual root biomass score (H^2^ = 0.103 and h^2^ = 0.028).

### Introgression lines displayed similar above-ground traits and a full range of root configurations

**Fig. 5:**
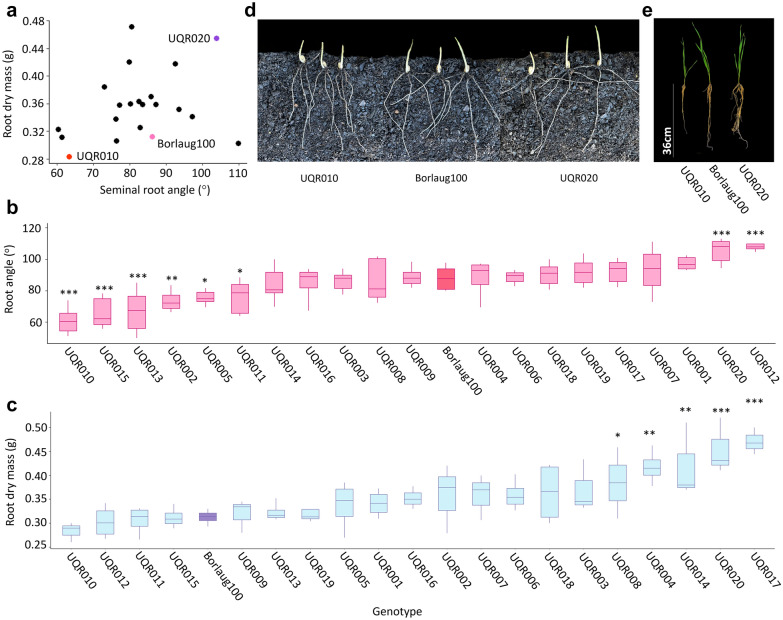
Phenotypes of the Borlaug100 introgression lines (BC_2_F_4_:F_5_) with modified root systems for seminal root angle and root biomass. **a** Scatter plot showing the 20 introgression lines (BC_2_F_4_:F_5_), displaying a spectrum of root configurations in the elite background with different seminal root angle and root dry biomass compared to Borlaug100. The line UQR010 (red) has a similar root dry mass to Borlaug100 (pink), but narrower root angle. The line UQR020 (purple) displays a wider root angle with higher root dry biomass. **b** Boxplots representing the seminal root angle for each introgression line. **c** Boxplots representing root dry biomass for each introgression line. For seminal root angle and root biomass a Fisher-LSD test was performed to compare the means between the introgression lines and Borlaug100 to determine if there are significant differences for both root traits (*Significant at the *P* ≤ 0.05, **Significant at the *P* ≤ 0.01, ***Significant at the *P* ≤ 0.001). **d** Comparison of the seminal root angle between Borlaug100 and the introgression lines UQR010 (narrow) and UQR020 (wide). **e** Demonstration of introgression lines with modified root biomass; UQR020 (high) and UQR010 (low) compared with Borlaug100

The final set of 20 introgression lines displayed similar above-ground traits to the recurrent parent Borlaug100 in the field. Borlaug100 had an average height of 103 cm and reached flowering within 92 days, and the 20 selected introgression lines ranged in height from 98 to 100 cm and flowered between 90 and 96 days (Additional file 1: Table S3). The 20 introgression lines displayed a high degree of variation in root phenotypes under controlled conditions (Fig. 5a). Six lines displayed significantly narrower seminal root angle compared to recurrent parent Borlaug100. For example, the seminal root angle of UQR010 was 8° narrower than Borlaug100 (85.8° ± 5°; Fig. 5b). In contrast, two introgression lines (UQR020 and UQR012) displayed significantly wider seminal root angle phenotypes (~ 12.5°) in comparison to Borlaug100. A total of five lines displayed significantly higher root biomass compared to Borlaug100 (Fig. 5d). Of these lines, UQR020 carried the 5B QTL and produced ~ 35% more root biomass (420 ± 3 mg) than the recurrent parent (312 ± 4 mg). The QTL for high root biomass was successfully introgressed into 5 of the 20 lines (Additional file 1: Table S3).

## Discussion

The aim of this study was to develop and validate a non-destructive root screening and selection approach using SPS. This approach integrates phenotypic and MAS, along with speed breeding to directly screen, select and introgress multiple root traits simultaneously. It provides a useful framework to develop elite materials with modified root systems to accelerate root research and breeding goals. The elite wheat lines developed in this study provide valuable materials to study the value of seminal root angle and root biomass traits to improve yield in a range of production scenarios.

### Visual assessment of root and shoot traits

In this study, visual scores for root biomass were highly correlated with dry root biomass (r = 0.83), demonstrating that root biomass of young plants can be estimated in a non-destructive manner. Notably, visual root biomass scores were a reflection of the overall size of the root system at a specific point in time. Several mechanisms could contribute to root biomass accumulation, such as additional branching of seminal roots, thicker roots, early nodal root development or longer roots. Arifuzzaman and Rahman [30] reported visual assessment of root traits in rapeseed, specifically root vigour, and found a significant correlation with root dry weight (r = 0.55–0.60, *P* ≤ 0.0001). This suggests that root vigour scoring could also be a surrogate trait for root dry weight [30]. Furthermore, automatic image analysis techniques could be incorporated to replace visual assessment and eliminate variation associated with operator error.

The strong correlation observed in our study between root and shoot dry biomass (r = 0.73) suggests that selection applied to root biomass would indirectly influence shoot biomass. To enable more targeted manipulation of R:S ratio, shoot biomass data could be integrated into the selection procedure. Interestingly, wheat genotypes examined in this study also showed significant variation for R:S ratio. For example, the smallest R:S ratio was displayed by Suntop (0.75), and the highest was displayed by Mace (0.99). Thus, despite the correlation between root and shoot biomass, 25% of the variation in carbon partitioning above- and below-ground is independent and can be manipulated. This provides an opportunity to combine different root and shoot traits essential for breeding for a range of target environments.

### Single plant selection for root traits: opportunities and challenges

The SPS method reported in this study rapidly assembled different combinations of seminal root angle and root biomass into an elite genetic background through repeated cycles of backcrossing. Despite the strong selection pressure for plant height and a narrow flowering window in the field, the final set of introgression lines displayed a spectrum of root trait configurations. This highlights the effectiveness of bi-directional selection to maintain trait diversity and enable the identification of individual plants with desirable trait combinations. The study by Richard et al. [31] also reported bi-directional selection for seminal root angle, which shifted the population mean by 10°. From simple geometry it can be appreciated that even small changes in angle can result in a significant difference in spread of the root system at depth, assuming no physical barriers to root growth along the initial trajectory. These results highlight the effectiveness of phenotypic selection for root angle in early generations of population development and the ability to shift population means as a response to selection.

The low heritability of root traits measured on a single plant basis was somewhat expected. Traits controlled by many genes with minor effects have low heritability [32, 33], and root traits in particular, are variable and interact with their environment [13, 34]. Despite these challenges, the selection approach successfully modified the target root traits in the Borlaug100 background. To counter low heritability and support selection for root biomass, individual plants selected based on extreme phenotypes were also screened using KASP markers linked to a large effect QTL on chromosome 5B [27, 28]. Among the elite lines developed in this study, not all carried the high root biomass QTL. Those lacking the QTL likely carry additional unknown genes modulating root biomass. Therefore, by combining phenotypic and MAS, it provides the opportunity to identify individual plants that carry ‘good gene combinations’, which could involve both known and unknown genes [35].

In order to combine root traits into different configurations, it is essential for the target traits to be controlled by independent loci. While a weak relationship between root angle and root biomass was observed during population screening (r = 0.17, *P* = 0.07), the transgressive segregation and unique trait combinations in the resulting introgression lines (Fig. 5a) suggests the root traits are underpinned by multiple genes, some of which are independent. Ideally, trait relationships and their genetic controls should be considered prior to introgression activities to ensure a successful outcome. Insight from genetic studies can help determine optimal population sizes to screen and selection intensity to be applied each generation [36]. In this study, early segregating generations were screened for root traits, which enabled selection of individual plants carrying desirable root traits that were advanced to the next generation. To further improve confidence during the selection process, ‘within and between family selection’ [37] could be implemented. This involves selecting the best individuals from the best families, where family performance is based on the average phenotype displayed by individuals in that family [38].

### Applications for research and pre-breeding

The elite wheat lines with modified root traits provide valuable genetic materials to study the value of root traits to support yield in different environments and soil types. The lines with narrow root angle could support deeper root growth and could offer yield benefits under terminal drought conditions, particularly when soil moisture is available at depth [36, 39]. The root biomass plays a critical role for the crop partitioning of the assimilates. Thus, the potential trade-offs that are associated with different partitioning strategies, needs to be carefully evaluated in context of which resources are limiting yield [40].

Pre-breeding programs have focused on studying diversity panels and bi-parental mapping populations to discover root trait QTL, and as a result, have found it challenging to precisely quantify the value of specific root traits [41]. A major constraint of working at the population level is the segregation of above-ground developmental traits that affect the timing of water-use and carbon partitioning. Examining elite introgression lines in field experiments with minimal differences in shoot traits will enable a fairer comparison of root traits without results being compromised by major phenology differences. This targeted approach to validate root trait QTLs and trait value could help accelerate progress in wheat [41].

The approach reported in this study represents a useful ‘tool-kit’ that could be used to target different root traits in wheat (for example root length, rate of growth or root hairs), or could be adapted to modify root systems in other crops. We consider this to be a useful framework because it combines the use of phenotypic and marker-assisted selection, along with speed breeding to accelerate the breeding goal. Speed breeding protocols are now available for many long-day and short-day crop species [42, 43], and these rapid cycling systems are ideal for accelerating trait introgression and pre-breeding programs. Shoot traits could also be targeted using a similar approach and could be used to generate novel germplasm to study the interactions between above- and below-ground developmental components.

## Conclusions

This study reports a toolkit to rapidly modify root systems through SPS. The method avoids plant destruction and enables selection in early generations to identify plants with extreme root phenotypes that can be advanced or backcrossed. The approach was used to develop introgression lines with different root configurations, but similar plant height and flowering time to the recurrent parent. The elite wheat lines with modified root traits provide useful materials to assess the value of root traits for yield improvement in different environments and production systems, in a defined genetic background. The SPS approach provides a framework for researchers and plant breeders aiming to optimise root systems of future crop varieties.

## Supplementary Information

**Additional file 1: Fig. S1. **Exemplification of how the targeted root traits (seminal root angle and root biomass) were combined to develop wheat lines with different types of root systems (see Fig. 2b). **Fig. S2.** Backcrossing scheme for the development of elite wheat introgression lines combining seminal root angle and root biomass in different configurations. *Purple *boxes indicate the generations that were subjected to bi-directional selection for root traits using the SPS approach. The resulting BC_2_F_4_ lines were phenotyped for above-ground traits (plant height and flowering time) in the field. The BC_2_F_4:5_ lines were also characterised for seminal root traits in a replicated phenotyping experiment under controlled conditions to confirm differences in root traits compared to the respective recurrent parent. **Fig. S3.** Boxplots displaying **(a)** visual score and **(b)** root dry biomass for each genotype screened. **Table S1.** Summary of relevant traits of all the lines used to determine the feasibility of screening individual wheat plants for both seminal root angle and root biomass. Values are the mean of 6 replicates. QTL status: --/- = line carries the haplotypes associated with low root biomass (i.e. h1, h2 or h8 for haploblock b and h1 for haploblock a), whereas **++/+** = line carries both desirable haplotypes for high root biomass (i.e. h3 for haploblock b and h2 for haploblock a) [27, 28]. **Table S2.** Comparison between root:shoot (R:S) ratio means using the Fisher-LSD test for the panel of seventeen lines in Fig. 1d used for visual score for estimating root biomass non-destructively. The table displays significant differences between genotypes in R:S ratio (* Significant at the *P* ≤ 0.05; ** Significant at the *P* ≤ 0.01; ***Significant at the *P* ≤ 0.001). In this panel were included the recurrent parents Borlaug100 and the three donors (SW107, SW309 and SW388) used to develop the introgression lines with different root configurations. **Table S3. **Details of the selected Borlaug100 introgression lines, including generation (FGen), pedigree, field above-ground measurements (plant height, days to flowering) and controlled environment root phenotypes (seminal root angle and root dry biomass).

**Additional file 2.** Single plant selection shopping list.

## Data Availability

The datasets analysed during the current study are available from the corresponding authors on reasonable request.
