# Supplementary material for: A toolkit to rapidly modify root systems through single plant selection
Source: Plant Methods. 2022 Jan 10;18:2. doi: 10.1186/s13007-021-00834-2 (PMC8750989; doi:10.1186/s13007-021-00834-2)
Supplement: Supplementary file 1 — Additional file 1: Fig. S1. Exemplification of how the targeted root traits (seminal root angle and root biomass) were combined to develop wheat lines with different types of root systems (see Fig. 2b). Fig. S2. Backcrossing scheme for the development of elite wheat introgression lines combining seminal root angle and root biomass in different configurations. Purple boxes indicate the generations that were subjected to bi-directional selection for root traits using the SPS approach. The resulting BC2F4 lines were phenotyped for above-ground traits (plant height and flowering time) in the field. The BC2F4:5 lines were also characterised for seminal root traits in a replicated phenotyping experiment under controlled conditions to confirm differences in root traits compared to the respective recurrent parent. Fig. S3. Boxplots displaying (a) visual score and (b) root dry biomass for each genotype screened. Table S1. Summary of relevant traits of all the lines used to determine the feasibility of screening individual wheat plants for both seminal root angle and root biomass. Values are the mean of 6 replicates. QTL status: --/- = line carries the haplotypes associated with low root biomass (i.e. h1, h2 or h8 for haploblock b and h1 for haploblock a), whereas ++/+ = line carries both desirable haplotypes for high root biomass (i.e. h3 for haploblock b and h2 for haploblock a) [27, 28]. Table S2. Comparison between root:shoot (R:S) ratio means using the Fisher-LSD test for the panel of seventeen lines in Fig. 1d used for visual score for estimating root biomass non-destructively. The table displays significant differences between genotypes in R:S ratio (* Significant at the P ≤ 0.05; ** Significant at the P ≤ 0.01; ***Significant at the P ≤ 0.001). In this panel were included the recurrent parents Borlaug100 and the three donors (SW107, SW309 and SW388) used to develop the introgression lines with different root configurations. Table S3. Details of the selected Borlaug100 [file 13007_2021_834_MOESM1_ESM.docx]

**
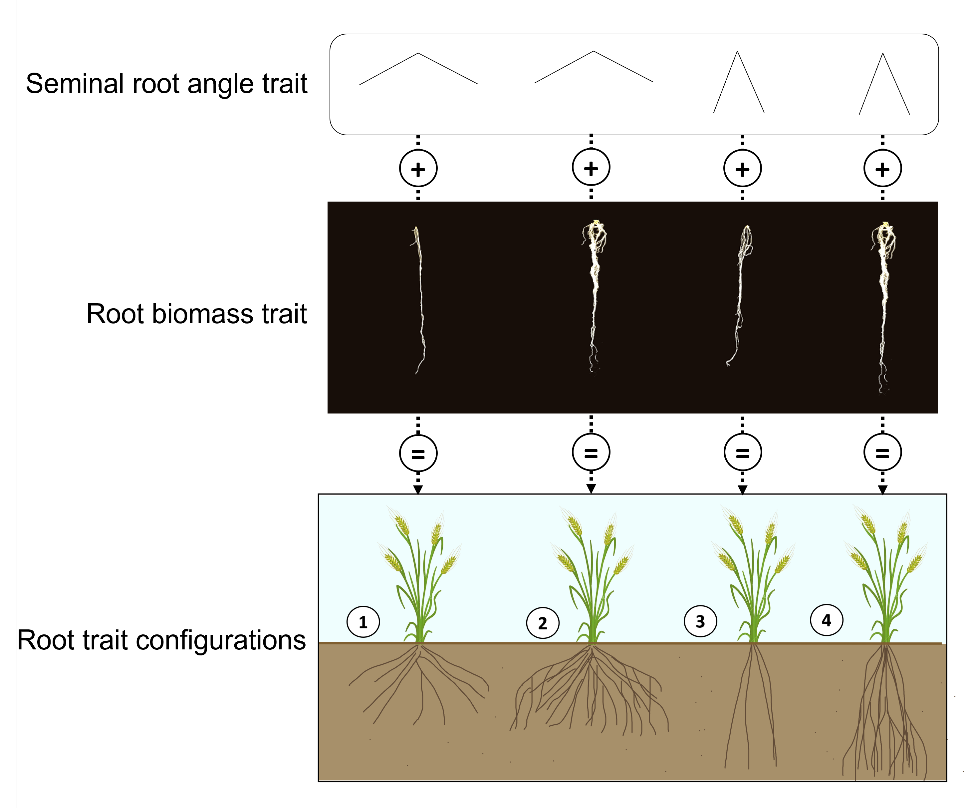
**

**Fig. S1.** Exemplification of how the targeted root traits (seminal root angle and root biomass) were combined to develop wheat lines with different types of root systems (see Fig. 2b).


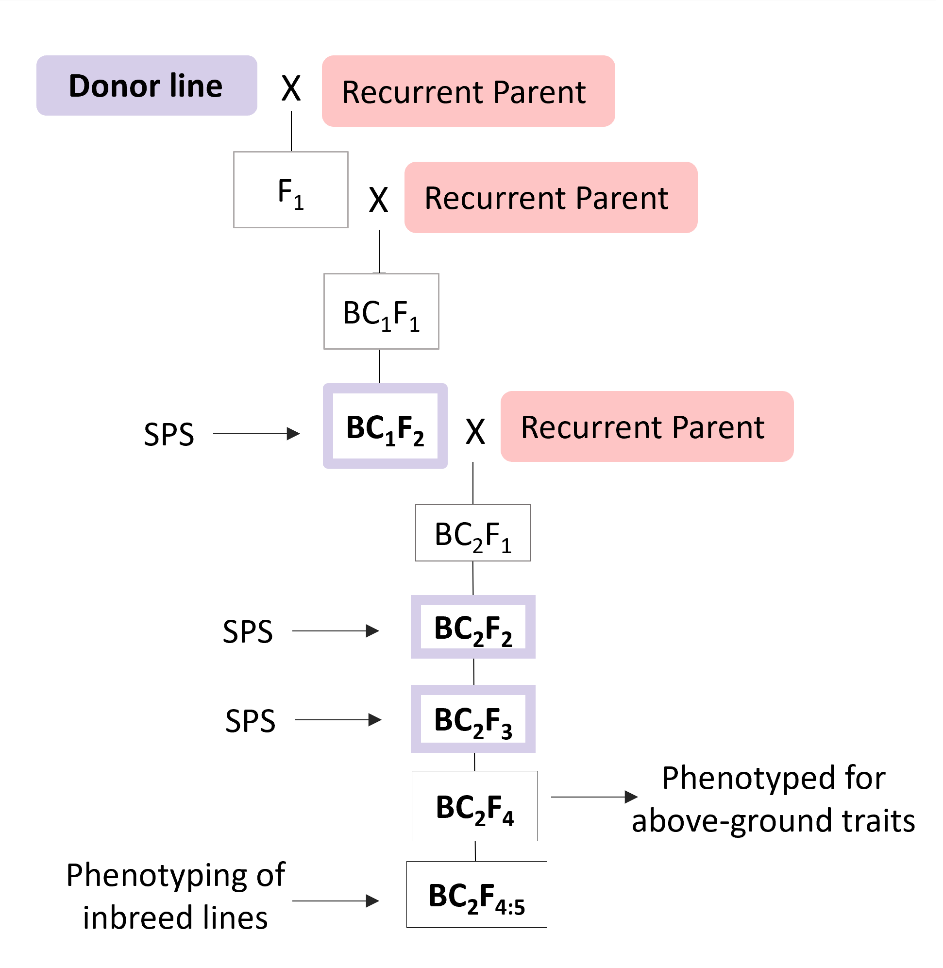


**Fig. S2.** Backcrossing scheme for the development of elite wheat introgression lines combining seminal root angle and root biomass in different configurations. *Purple* boxes indicate the generations that were subjected to bi-directional selection for root traits using the SPS approach. The resulting BC_2_F_4_ lines were phenotyped for above-ground traits (plant height and flowering time) in the field. The BC_2_F_4:5_ lines were also characterised for seminal root traits in a replicated phenotyping experiment under controlled conditions to confirm differences in root traits compared to the respective recurrent parent.

**
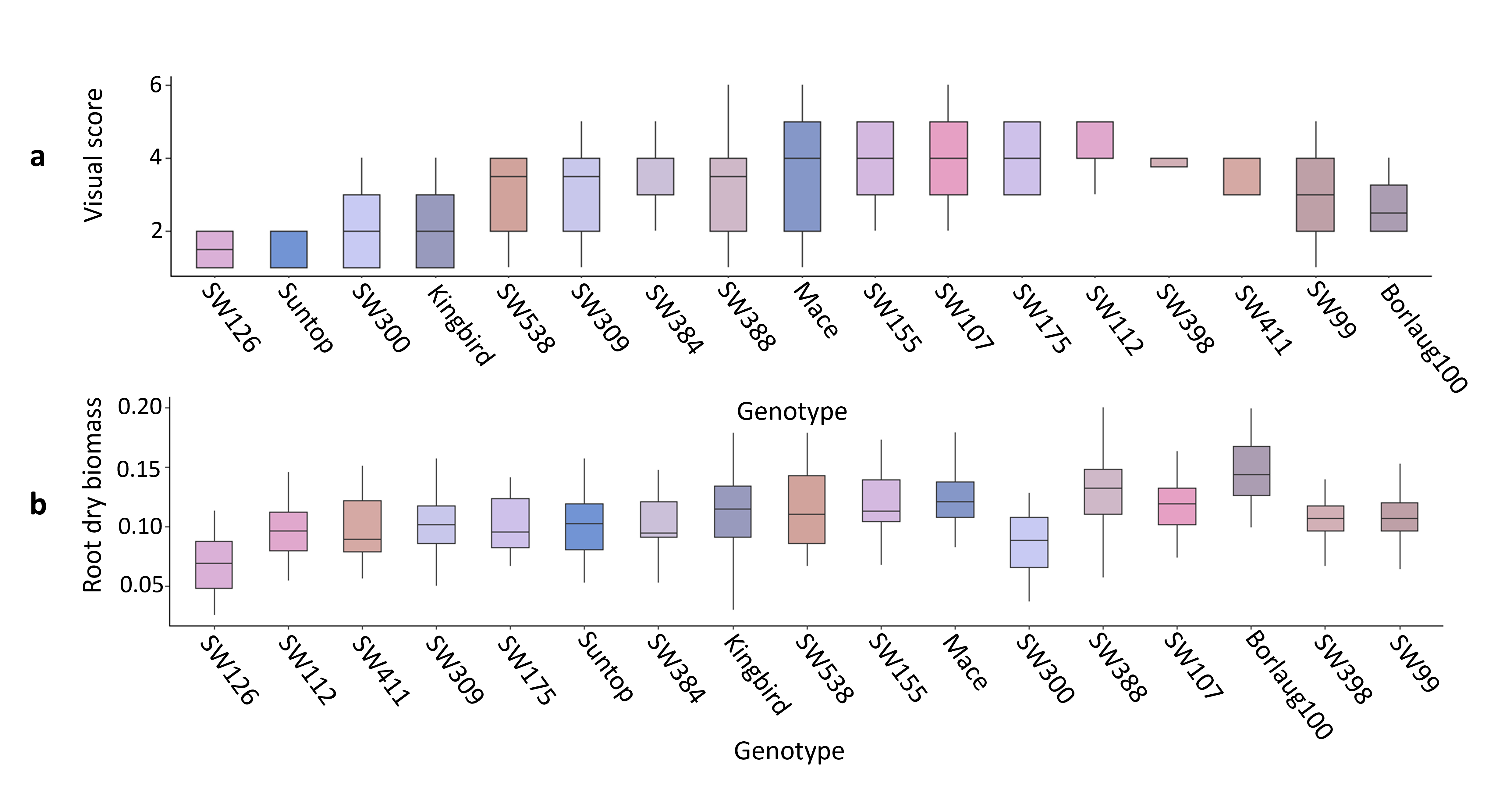
**

**Fig. S3.** Boxplots displaying **(a)** visual score and **(b)** root dry biomass for each genotype screened.

**Table S1.** Summary of relevant traits for all lines used to determine the feasibility of screening individual wheat plants for both seminal root angle and root biomass. Values are the mean of 6 replicates. QTL status: --/- = line carries the haplotypes associated with low root biomass (i.e. h1, h2 or h8 for haploblock b and h1 for haploblock a), whereas **++/+** = line carries both desirable haplotypes for high root biomass (i.e. h3 for haploblock b and h2 for haploblock a) [27;28].

| **Genotype** | **Seminal root angle** | **Root dry biomass** | **Shoot dry biomass** | **Root shoot ratio** | **QTL status** |
| --- | --- | --- | --- | --- | --- |
| SW107 | 63.9 | 0.1412 | 0.979 | 0.979 | ++/+ |
| SW112 | 66.1 | 0.0965 | 0.929 | 0.929 | - -/- |
| SW126 | 64.0 | 0.0675 | 0.831 | 0.831 | - -/- |
| SW155 | 62.1 | 0.1225 | 0.772 | 0.772 | - -/- |
| SW175 | 56.9 | 0.1010 | 0.872 | 0.872 | - -/- |
| SW300 | 69.3 | 0.1265 | 0.933 | 0.933 | - -/- |
| SW309 | 72.3 | 0.1001 | 0.76 | 0.76 | - -/- |
| SW384 | 55.3 | 0.1043 | 0.786 | 0.786 | - -/- |
| SW388 | 66.6 | 0.1271 | 0.753 | 0.753 | ++/+ |
| SW398 | 63.8 | 0.1043 | 0.862 | 0.862 | - -/- |
| SW411 | 65.5 | 0.0985 | 0.894 | 0.894 | - -/- |
| SW538 | 64.5 | 0.1205 | 0.957 | 0.957 | - -/- |
| SW99 | 56.6 | 0.1057 | 0.799 | 0.799 | - -/- |
| Borlaug100 | 89.6 | 0.1555 | 0.864 | 0.864 | - -/- |
| Kinbgbird | 79.3 | 0.1109 | 0.769 | 0.769 | - -/- |
| Mace | 104.5 | 0.1241 | 0.994 | 0.994 | - -/- |
| Suntop | 96.1 | 0.1014 | 0.769 | 0.769 | - -/- |

**Table S2.** Comparison between root:shoot (R:S) ratio means using the Fisher-LSD test for the panel of seventeen lines in Fig. 1d used for non-destructive visual assessment of root biomass. The table displays significant differences between genotypes in R:S ratio (* Significant at the *P* ≤ 0.05; ** Significant at the *P* ≤ 0.01; ***Significant at the *P* ≤ 0.001). The panel includes recurrent parent Borlaug100 and the three donor lines (SW107, SW309 and SW388) used to develop the introgression lines with different root configurations.

| **Comparison** | **p-value** | **Significance** |
| --- | --- | --- |
| Borlaug100 - Kinbgbird | 0.0858 | ns |
| Borlaug100 - Mace | 0.0195 | * |
| Borlaug100 - Suntop | 0.0413 | * |
| Borlaug100 - SW107 | 0.0398 | * |
| Borlaug100 - SW112 | 0.2422 | ns |
| Borlaug100 - SW126 | 0.5526 | ns |
| Borlaug100 - SW155 | 0.4899 | ns |
| Borlaug100 - SW175 | 0.888 | ns |
| Borlaug100 - SW300 | 0.085 | ns |
| Borlaug100 - SW309 | 0.0604 | ns |
| Borlaug100 - SW384 | 0.1553 | ns |
| Borlaug100 - SW388 | 0.0436 | * |
| Borlaug100 - SW398 | 0.9628 | ns |
| Borlaug100 - SW411 | 0.5906 | ns |
| Borlaug100 - SW538 | 0.0951 | ns |
| Borlaug100 - SW99 | 0.0828 | ns |
| Kinbgbird - Mace | 1.00E-04 | *** |
| Kinbgbird - Suntop | 0.7313 | ns |
| Kinbgbird - SW107 | 2.00E-04 | *** |
| Kinbgbird - SW112 | 0.004 | ** |
| Kinbgbird - SW126 | 0.2601 | ns |
| Kinbgbird - SW155 | 0.3031 | ns |
| Kinbgbird - SW175 | 0.0632 | ns |
| Kinbgbird - SW300 | 7.00E-04 | *** |
| Kinbgbird - SW309 | 0.8722 | ns |
| Kinbgbird - SW384 | 0.7656 | ns |
| Kinbgbird - SW388 | 0.7625 | ns |
| Kinbgbird - SW398 | 0.0983 | ns |
| Kinbgbird - SW411 | 0.0243 | * |
| Kinbgbird - SW538 | 8.00E-04 | *** |
| Kinbgbird - SW99 | 0.9866 | ns |
| Mace - Suntop | 0 | *** |
| Mace - SW107 | 0.7967 | ns |
| Mace - SW112 | 0.2409 | ns |
| Mace - SW126 | 0.0035 | ** |
| Mace - SW155 | 0.0026 | ** |
| Mace - SW175 | 0.0281 | * |
| Mace - SW300 | 0.5531 | ns |
| Mace - SW309 | 0 | *** |
| Mace - SW384 | 2.00E-04 | *** |
| Mace - SW388 | 0 | *** |
| Mace - SW398 | 0.0184 | * |
| Mace - SW411 | 0.0715 | ns |
| Mace - SW538 | 0.5016 |  |
| Mace - SW99 | 1.00E-04 | *** |
| Suntop - SW107 | 1.00E-04 | *** |
| Suntop - SW112 | 0.0015 | ** |
| Suntop - SW126 | 0.1453 | ns |
| Suntop - SW155 | 0.1734 | ns |
| Suntop - SW175 | 0.0293 | * |
| Suntop - SW300 | 2.00E-04 | *** |
| Suntop - SW309 | 0.8538 | ns |
| Suntop - SW384 | 0.5234 | ns |
| Suntop - SW388 | 0.9646 | ns |
| Suntop - SW398 | 0.0484 | * |
| Suntop - SW411 | 0.0102 | * |
| Suntop - SW538 | 2.00E-04 | *** |
| Suntop - SW99 | 0.7438 | ns |
| SW107 - SW112 | 0.3664 | ns |
| SW107 - SW126 | 0.0084 | ** |
| SW107 - SW155 | 0.0063 | ** |
| SW107 - SW175 | 0.0552 | ns |
| SW107 - SW300 | 0.7398 | ns |
| SW107 - SW309 | 1.00E-04 | *** |
| SW107 - SW384 | 6.00E-04 | *** |
| SW107 - SW388 | 1.00E-04 | *** |
| SW107 - SW398 | 0.0375 | * |
| SW107 - SW411 | 0.1268 | ns |
| SW107 - SW538 | 0.6837 | ns |
| SW107 - SW99 | 2.00E-04 | *** |
| SW112 - SW126 | 0.0783 | ns |
| SW112 - SW155 | 0.0633 | ns |
| SW112 - SW175 | 0.3035 | ns |
| SW112 - SW300 | 0.5701 | ns |
| SW112 - SW309 | 0.0024 | ** |
| SW112 - SW384 | 0.0098 | ** |
| SW112 - SW388 | 0.0015 | ** |
| SW112 - SW398 | 0.2288 | ns |
| SW112 - SW411 | 0.5273 | ns |
| SW112 - SW538 | 0.616 | ns |
| SW112 - SW99 | 0.0038 | ** |
| SW126 - SW155 | 0.923 | ns |
| SW126 - SW175 | 0.4626 | ns |
| SW126 - SW300 | 0.0211 | * |
| SW126 - SW309 | 0.1982 | ns |
| SW126 - SW384 | 0.4073 | ns |
| SW126 - SW388 | 0.1534 | ns |
| SW126 - SW398 | 0.5885 | ns |
| SW126 - SW411 | 0.258 | ns |
| SW126 - SW538 | 0.0239 | * |
| SW126 - SW99 | 0.2531 | ns |
| SW155 - SW175 | 0.4059 | ns |
| SW155 - SW300 | 0.0164 | * |
| SW155 - SW309 | 0.2339 | ns |
| SW155 - SW384 | 0.4641 | ns |
| SW155 - SW388 | 0.1831 | ns |
| SW155 - SW398 | 0.5244 | ns |
| SW155 - SW411 | 0.2196 | ns |
| SW155 - SW538 | 0.0186 | * |
| SW155 - SW99 | 0.2953 | ns |
| SW175 - SW300 | 0.1132 | ns |
| SW175 - SW309 | 0.0436 | * |
| SW175 - SW384 | 0.1184 | ns |
| SW175 - SW388 | 0.0309 | * |
| SW175 - SW398 | 0.8525 | ns |
| SW175 - SW411 | 0.6912 | ns |
| SW175 - SW538 | 0.1263 | ns |
| SW175 - SW99 | 0.0609 | ns |
| SW300 - SW309 | 4.00E-04 | *** |
| SW300 - SW384 | 0.0018 | ** |
| SW300 - SW388 | 2.00E-04 | *** |
| SW300 - SW398 | 0.08 | ns |
| SW300 - SW411 | 0.2331 | ns |
| SW300 - SW538 | 0.9428 | ns |
| SW300 - SW99 | 6.00E-04 | *** |
| SW309 - SW384 | 0.6463 | ns |
| SW309 - SW388 | 0.8876 | ns |
| SW309 - SW398 | 0.0701 | ns |
| SW309 - SW411 | 0.0159 | * |
| SW309 - SW538 | 4.00E-04 | *** |
| SW309 - SW99 | 0.8854 | ns |
| SW384 - SW388 | 0.5484 | ns |
| SW384 - SW398 | 0.174 | ns |
| SW384 - SW411 | 0.0504 | ns |
| SW384 - SW538 | 0.0021 | ** |
| SW384 - SW99 | 0.7529 | ns |
| SW388 - SW398 | 0.0511 | ns |
| SW388 - SW411 | 0.0108 | * |
| SW388 - SW538 | 2.00E-04 | *** |
| SW388 - SW99 | 0.7753 | ns |
| SW398 - SW411 | 0.5627 | ns |
| SW398 - SW538 | 0.0896 | ns |
| SW398 - SW99 | 0.095 | ns |
| SW411 - SW538 | 0.2572 | ns |
| SW411 - SW99 | 0.0233 | * |
| SW538 - SW99 | 7.00E-04 | *** |

**Table S3.** Details of the selected Borlaug100 introgression lines, including generation (FGen), pedigree, field above-ground measurements (plant height, days to flowering) and controlled environment root phenotypes (seminal root angle and root dry biomass).

| Genotype | FGen | Pedigree | PH (cm) | DTF (days) | RA (°) | RDB (g) | QTL status |
| --- | --- | --- | --- | --- | --- | --- | --- |
| UQR001 | BC_2_F_4:_F_5_ | SW388*3/Borlaug100 | 93 | 94 | 89.6 | 343 | - -/- |
| UQR002 | BC_2_F_4:_F_5_ | SW388*3/Borlaug100 | 90 | 94 | 84.8 | 325 | **++/+** |
| UQR003 | BC_2_F_4:_F_5_ | SW388*3/Borlaug100 | 103 | 88 | 85.6 | 371 | **++/+** |
| UQR004 | BC_2_F_4:_F_5_ | SW388*3/Borlaug100 | 100 | 91 | 90.3 | 418 | - -/- |
| UQR005 | BC_2_F_4:_F_5_ | SW107*3/Borlaug100 | 104 | 90 | 76.2 | 340 | - -/- |
| UQR006 | BC_2_F_4:_F_5_ | SW388*3/Borlaug100 | 104 | 94 | 95.2 | 360 | - -/- |
| UQR007 | BC_2_F_4:_F_5_ | SW388*3/Borlaug100 | 98 | 92 | 87.4 | 320 | - -/- |
| UQR008 | BC_2_F_4:_F_5_ | SW388*3/Borlaug100 | 98 | 92 | 89.7 | 360 | - -/- |
| UQR009 | BC_2_F_4:_F_5_ | SW107*3/Borlaug100 | 90 | 94 | 80.1 | 360 | - -/- |
| UQR010 | BC_2_F_4:_F_5_ | SW107*3/Borlaug100 | 96 | 99 | 77.5 | 315 | **++/+** |
| UQR011 | BC_2_F_4:_F_5_ | SW107*3/Borlaug100 | 107 | 97 | 77.6 | 308 | - -/- |
| UQR012 | BC_2_F_4:_F_5_ | SW107*3/Borlaug100 | 96 | 97 | 98.3 | 303 | - -/- |
| UQR013 | BC_2_F_4:_F_5_ | SW107*3/Borlaug100 | 105 | 92 | 76.0 | 323 | - -/- |
| UQR014 | BC_2_F_4:_F_5_ | SW107*3/Borlaug100 | 78 | 89 | 86.2 | 375 | **++/+** |
| UQR015 | BC_2_F_4:_F_5_ | SW107*3/Borlaug100 | 100 | 93 | 65.2 | 313 | - -/- |
| UQR016 | BC_2_F_4:_F_5_ | SW107*3/Borlaug100 | 98 | 88 | 79.8 | 403 | - -/- |
| UQR017 | BC_2_F_4:_F_5_ | SW107*3/Borlaug100 | 96 | 102 | 86.5 | 430 | - -/- |
| UQR018 | BC_2_F_4:_F_5_ | SW388*3/Borlaug100 | 102 | 89 | 88.9 | 365 | - -/- |
| UQR019 | BC_2_F_4:_F_5_ | SW388*3/Borlaug100 | 107 | 95 | 92.0 | 325 | - -/- |
| UQR020 | BC_2_F_4:_F_5_ | SW388*3/Borlaug100 | 103 | 94 | 94.8 | 420 | **++/+** |

(PH) Plant height, (DTF) Days to flowering, (RA) Seminal root angle, (RDB) Root dry biomass, (QTL status) --/- = line carries the haplotypes associated with low root biomass (i.e. h1, h2 or h8 for haploblock b and h1 for haploblock a), whereas **++/+** = line carries both desirable haplotypes for high root biomass (i.e. h3 for haploblock b and h2 for haploblock a) [27;28].
