# Supplementary material for: A toolkit to rapidly modify root systems through single plant selection
Source: Plant Methods. 2022 Jan 10;18:2. doi: 10.1186/s13007-021-00834-2 (PMC8750989; doi:10.1186/s13007-021-00834-2)
Supplement: Supplementary file 2 — Additional file 2. Single plant selection shopping list. [file 13007_2021_834_MOESM2_ESM.pdf]

Additional file 2

Single plant selection (SPS) shopping list:

Seminal root angle screen *(Richard et al., 2015)*

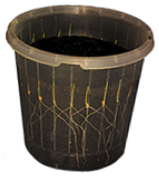

Clear pot 4 L

- Clear pot 4 L (ANOVApot®, 200 mm top diameter, 190 mm height)

- 4 L black pots (ANOVApot®, 200 mm diameter, 190 mm height)

- Tags

- Marker pen

- Pine bark potting media (70% composted pine bark 0-5 mm, 30% coconut peat, pH 6.35, EC = 650 ppm, nitrate = 0, ammonium < 6 ppm and phosphorus = 50 ppm)

- Camera/phone (ImageJ software to analyse seminal root angle images)

- Tweezers

Semi-hydroponic sand-based system

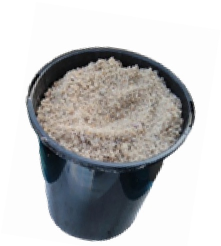

1.4 L ANOVA pot

- 1.4 L ANOVA pot (ANOVApot®, 137 mm diameter, 140 mm height)

- Tags

- Marker pen

- Hydroponic solution:

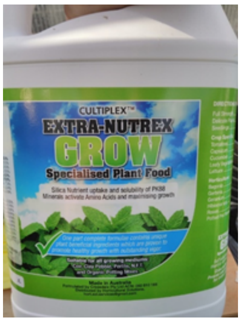

Nutrients details: Total Nitrogen (N) 20.0 %, As Amide (5.0%), As Nitrate (9.0%), As Ammonium (6.0%), Total Phosphorus (P) 8%, Total Potassium (K) 18%, Calcium (Ca) 7.3%, Magnesium (Mg) 2.4% , Sulfur (S) 0.9%, (Fr) as EDTA 0.22%, (Mn) as EDTA 0.16%, (Zn) as Amino Acid 0.05%, (Cu) as Amino Acid 0.006%, *(Mo) as Molybdenum 0.009%, *(B) as Boric Acid 0.06%, (Co) as Amino Acid 0.005%, (Si) as Silicic Acid 2.2%, PK88 as, Colloidal Minerals 2.2%. * Present as inorganic form, Dry weight %w/w.

We used the brand Cultiplex Extra Nutrex Grow 5L

- Coarse washed sand (particle size ranging 0.075-4.75 mm)

- Capillary mat (3mm thick to put inside the container, ProFab® Non Woven (globalsynthetics.com.au))

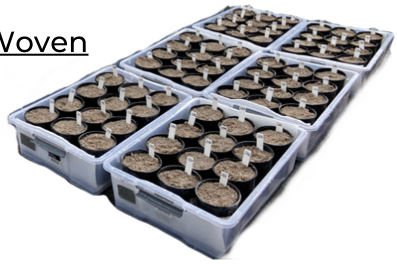

- Containers (65 cm length and 35 cm width with a capacity of 36 L)

- Watering can (9L)

- Disposable transfer pipette pasteur or small container to measure hydroponic solution
